# Applications and Immunological Effects of Quantum Dots on Respiratory System

**DOI:** 10.3389/fimmu.2021.795232

**Published:** 2022-01-06

**Authors:** Laibin Ren, Lingwei Wang, Markus Rehberg, Tobias Stoeger, Jianglin Zhang, Shanze Chen

**Affiliations:** ^1^ Institute of Respiratory Diseases, Shenzhen People’s Hospital, Jinan University, Shenzhen, China; ^2^ The First Affiliated Hospital, Southern University of Science and Technology, Shenzhen, China; ^3^ Comprehensive Pneumology Center, Institute of Lung Biology and Disease, Helmholtz Center Munich, German Research Center for Environmental Health, Neuherberg and Member of the German Center for Lung Research, Munich, Germany; ^4^ Department of Dermatology, Shenzhen People’s Hospital, Jinan University, Shenzhen, China

**Keywords:** quantum dot, respiratory system, biomedical applications, cytotoxicity, immunological effects

## Abstract

Quantum dots (QDs), are one kind of nanoscale semiconductor crystals with specific electronic and optical properties, offering near-infrared mission and chemically active surfaces. Increasing interest for QDs exists in developing theranostics platforms for bioapplications such as imaging, drug delivery and therapy. Here we summarized QDs’ biomedical applications, toxicity, and immunological effects on the respiratory system. Bioapplications of QDs in lung include biomedical imaging, drug delivery, bio-sensing or diagnosis and therapy. Generically, toxic effects of nanoparticles are related to the generation of oxidative stresses with subsequent DNA damage and decreased lung cells viability *in vitro* and *in vivo* because of release of toxic metal ions or the features of QDs like its surface charge. Lastly, pulmonary immunological effects of QDs mainly include proinflammatory cytokines release and recruiting innate leukocytes or adaptive T cells.

## Introduction

Quantum dots (QDs), one of the extensively studied nanoparticle material forms, have specific optical, photochemical, and electronic properties. The quantum confinement effect was firstly reported by Ekimov and Onushchenko in 1981, when they observed a size effect on the absorption characteristics of CuCl crystals dispersed in silicate glasses (1). The application of QDs on biological system for bioimaging started in1998 (2).

Generally, traditional organic label dyes have no ability of producing the near-infrared emission highly desired for biological imaging because of its high tissue penetration (low light scattering and absorption), and for this reason tunable optical QDs have gained utmost interests. Depending on specific properties of the material, different kinds of QDs could even be excited by the very same wavelength light, and their narrow emission stripes could be detected in parallel at individual wavelengths, allowing the conduction of different assays simultaneously. The compositions, shell thickness, and size of QDs determine the fluorescence bands (3). Generally QDs, like CdTeS, PbS, and HgTe, consist of elements such as Cd, Pb, and Hg from the II–VI, III–IV and IV–VI groups of the periodic table. Additionally, ternary I–III–VI elements (such as Ag, Cu, and Se)-related QDs like Ag2S, CuInS2, and CdZnSe have been developed (4). Besides, massive research regarding QDs has shifted to new emerging materials. Among them silicon- and carbon-based QDs have attracted great attention. Especially, the carbon-based QDs, namely, carbon dots, carbon nanotube dots, and graphene QDs have characteristics such as improved biocompatibility, nontoxicity or low toxicity, eco-friendly, stable and photobleaching-resistant compared to conventional QDs (5, 6). It was reported that the distribution of carbon-based QDs in the organ is related to its volume size: the smaller, the harder to clear. Besides, the surface charge of QDs affects their distribution: charged QDs are more capable of protein adsorption, accumulating in organs like liver. On the contrary, the neutral ones without protein adsorption are safely removed by renal filtration (7).

## Bioapplications of QDs in Lung

### Pulmonary System Related Bioimaging

Though bio-imaging with fluorescence has been employed in cells or animal for decades, broader clinical applications are limited for the visible light poor transmission through biological tissues, which promoted scientists to employ the optical window of Near Infrared (NIR) to carry out deep-tissue optical imaging (8, 9). Many formulations of QDs for bio-imaging lung cell signaling, tissue structures or related lung diseases in respiratory system are currently available in the literature.

Overcoming traditional chest radiology by radiation-free, noninvasive imaging is an important field in nanotechnology. DNA methylation is an essential part of human epigenetic modifications. The abnormal patterns of DNA methylation are tightly connected with lots of cancers or genetic diseases of liver, colon, and lung (10). Wang et al. reported that fluorescence resonance energy transfer (FRET) based on QDs mediated by tricyclic ligation chain reaction (LCR) were employed to image and examine DNA methylation in H157 non-small cell lung cancer cells, detecting DNA methylation with single 5-methylcytosine resolution low to 1.0 aM and a 7-order dynamic magnitude scope, which held great potential for precisely epigenetically evaluating lung cancers (11).

As QDs could in real-time image and reflect biological molecular activities in cells, some attempts have been taken to study lung cellular signaling pathways. In lung vascular cell adhesion molecule-1 (VCAM) is central to lung inflammation because it facilitates recruiting and anchoring phagocytes to the pulmonary endothelium, potentially aggravating endothelial damages and eventual pulmonary dysfunction (12). NADPH oxidase 2 (Nox2) is the major source of inflammation-associated reactive oxygen species (ROS) production. Orndorff et al. showed that endothelial Nox2 induced VCAM expression associated with lung inflammation *in vivo* through functionalizing fluorescent QDs with antibodies toward VCAM to detect its expression in a mouse model, demonstrates the relationship between Nox2 and VCAM during lung inflammation (13).

Light microscopic imaging of blood vessels is a good way to observe the hemodynamics of lungs under normal or pathologic conditions. Saitoh et al. captured precise peripheral pulmonary alveoli blood flow time-courses by injecting glutathione-decorated QDs into heart right ventricles and at different time-points performing *in vivo* cryotechnique (IVCT) in normal or abnormal lung stages (acute pulmonary hypertension mouse model) (14), thereby facilitating the investigation of mice lung microvascular hemodynamics and the altered structures.

Several reports attempted to image lung tumor-related markers *in vitro* or *in vivo* by employing QDs. Liu et al. produced “Affibody” QDs (AF-QDs) to bio-image the human epidermal growth factor receptor type 2 (HER2) in human pulmonary tumor cells. The approach avoided complicated chemical conjugation process and demonstrated to be a promising way of fluorescent nanoprobes for imaging cancer targets (15). Xue et al. employed CdTeS QDs decorated by folate–polyethylene glycol (FA–PEG) to image the overexpressed folate receptor (FR) in the tumors, demonstrating good biocompatibility, excellent specificity, and sensitivity for tumors imaging. Su et al. reported iodine doped carbon dots conjugated with cetuximab as a dual fluorescent/CT probe for bioimaging lung cancer cells epidermal growth factor receptor (EGFR) (16). Additionally, QDs immunofluorescence histochemistry (QDs-IHC) was employed to detect EGFR mutant, RRM2 and Bcl2, and Monocarboxylate transporter 4 (MCT-4) in non-small cell lung cancer patients (17).

Moreover, QDs also have been used to image and detect lung-related viral infections. Using a three-dimensional single-particle tracking technique (SPT) and through labeling avian influenza H9N2 virus with QDs, Wang et al. found that the sialic acid receptors were highly consistent with the number of influenza virus in human bronchial epithelial (HBE) cells, indicating sialic acid receptors may facilitate monitoring the situation that avian influenza viruses infected humans beings (18). Furthermore, by *in vivo* labeling H5N1 pseudotype of avian influenza virus with QDs, Pan et al. found that QD-labeled H5N1p showed sustained and bright fluorescent intensity in mice pulmonary tissues, enabling them to observe respiratory viral infection noninvasively and in real-time (19). Importantly, Gorshkov et al. produced a probe (fluorescent QDs-conjugated recombinant Spike receptor binding domain which could bind to Angiotensin Converting Enzyme 2 (ACE2)) for tracking SARS-CoV-2 virus. By employing the probe, they found the probe immediately bound on the surface of ACE2-GFP-transfected cells with subsequent endocytosis (20).

### Drug Delivery Into Lung Tumors

QDs are desired candidates as drug nano-platforms because they can be part of a more complex architecture or as the main carrier. Currently, trials on the application of QDs for drug delivery in the respiratory system mainly focused on pulmonary tumors.

5-Fluorouracil (5FU), an analogue of pyrimidine inhibiting cell metabolism, is a widely employed chemotherapy drug in cancer treatment. Duman et al. developed PEGylated Ag_2_S QDs which were decorated with Cetuximab and carried with 5-fluorouracil (5FU) (an anticancer drug). PEGylated Ag_2_S QDs demonstrated effectively and selectively delivering 5FU into A549 cells with subsequently significantly increased apoptosis, and also overcame better the cell protective effect of better 5FU-induced autophagy (21).

The folate receptor (FR), highly overexpressing on human pulmonary cancer cells surface, represents another potential candidate for targeted tumor treatment. Ruzycka−Ayoush et al. showed that Ag–In–Zn–S QDs nanocrystals which were decorated with L−cysteine, 11−mercaptoundecanoic acid (MUA), and lipoic acid modified with folic acid (FA) can be employed as a good approach for engaging doxorubicin (DOX) to FRs in A549. The QD–MUA–FA–DOX complex had a great genotoxicity and cytotoxicity, and also inhibited the migration of A549 significantly (22).

Cai et al. presented NH_2_-ZnO QDs with hyaluronic acid (HA) decorated with the dicarboxyl-terminated PEG specifically bound to cancer cells glycoprotein CD44. DOX were introduced to PEG modified ZnO QDs decorated with PEG *via* covalent interactions and metal–DOX complex. After uptake, the pH-sensitive QDs dissolved and released Zn^2+^ ion into the endosome and lysosome, followed by a controlled DOX releasing and the metal–drug complex dissociating (23). Importantly, the results showed that Zn^2+^ preferentially killed the tumor cells but had little impact on the healthy control cells.

The development of efficient combination therapy has drawn great attention in the oncotherapy field. Based on QDs nanoparticles, Li et al. delivered small interfering RNA (siRNA), paclitaxel, carboplatin, and doxorubicin for targeting lung tumor. QD nanocarriers delivering Bcl-2-targeted siRNA with other anticancer drugs not only induced greatly higher inhibition in A549 viability than single but furthermore enabled the real-time bioimaging of the delivery of the medicants and release by employing the special fluorescence characteristics (24).

Chronic obstructive pulmonary disease is a nonmalignant but intractable ill condition, manifested by airway obstruction and the increase of sticky mucus layers. Accordingly, QDs material with mucus-penetrating ability offered a novel approach to therapeutically give medicants. Li et al. reported that black phosphorus QDs (BPQDs) modified with PEG-decorated chitosan nano-particle with amikacin, which facilitated deep penetration of nano-vehicles into the mucus layer. The rapid degradation of BPQDs promoted dissociation of PEGylated QDs, accelerated release of amikacin, and eventually destroyed the biofilms (25).

### Biosensor and Diagnosis of Lung Tumors

Based on QDs unique light properties, recently the new relevant molecules detection and quantification strategies have arisen. Currently, developing and exploring novel QDs diagnosis methods in respiratory system mainly focused on lung cancers.

For EGFRs overexpressed in the lung cancer cells, Chen et al. developed a novel DNA electrochemiluminescence (ECL) sensor combining with CuZnInS QDs and gold-nanoparticles to detect highly sensitively EGFR gene. The range of target DNA concentration was from 0.05 to 1 nmol/L, and the detection limit reached low enough to 0.0043 nmol/L (26).

Silencing or decreasing tumor suppressor genes expressions always helps the initiation and progression of cancers (27), and DNA methylation is tightly related to the initiation of cancers. Ma et al. utilized the QDs-based FRET nanosensor technology to detect the tumor suppressor genes—protocadherin gamma subfamily B, 6 (PCDHGB6), Homeobox A9 (HOXA9) and Ras association domain family 1 isoform A (RASSF1A)-promoters methylation in non-small-cell lung carcinoma (NSCLC) early-stage specimens or noninvasive bronchial brushing tissues. The method could identify pulmonary tumor tissue samples and noninvasive bronchial brushing tissues from healthy controls with an excellent sensitivity of 92 and 80% respectively (28).

CYFRA 21-1 (a cytokeratin 19 fragment) is part of intermediate filament proteins stabilizing epithelial cells. Its expression on various epithelial cells makes it a useful biomarker in lung or other organ cancers (29). Several studies attempted to develop QDs related methods for detecting CYFRA 21-1 for helping diagnose lung cancer. Firstly, Chen et al. reported that a novel lateral flow test strips (QPs-LFTS) system based on polystyrene QDs particles was generated to examine human serum carcinoembryonic antigen (CEA) and CYFRA 21-1 simultaneously. The limit of detection for CEA or CYFRA 21-1 was 0.35 or 0.16 ng/ml respectively, indicating the system is highly efficient enough to be employed for the early screening and prognosis of lung cancer patients (30). Also, Meng et al. reported that molybdenum oxide QDs (MoOx QDs) were generated in one-pot manner and employed as a versatile probe in an ECL immunoassay of CYFRA21-1 as a model analyte (31). Besides, Alarfaj reported a different way of detecting CYFRA 21-1 that the green synthesized carbon QDs conjugated ZnO nanocomposite using Citrus lemon pericarp quickly determinate human serum CYFRA 21-1 antigen (32). Additionally, Liu et al, presented a method by combining the suspension and planar microarray formats in a single polydimethylsiloxane layer. On the basis of the target proteins, they formed a sandwich structure between the QD probes and the magnetic beads by specific antigen–antibody interactions, which could be used for simultaneous detecting pulmonary tumor biomarkers (CEA, CYFRA21-1 and neuron-specific enolase) with a broad linear dynamic scope and a low detection limit (33).

### Therapy Against Lung Tumors, Infection and Pulmonary Arterial Hypertension

QDs have been shown in various applications from the treatment of lung tumors to kill pulmonary infection-related bacteria and also alleviate pulmonary arterial hypertension.

In this context, Sun et al. reported that CdSe/ZnS-3-mercaptopropionic acid and CdSe/ZnS-glutathione QDs could inhibit the expressions of P-glycoprotein gene and protein accounting for multidrug resistance of lung cancer cells by inducing miR-185 and miR-34b, indicating miR-185 and miR-34b could be also interesting and potential targets for lung cancer treatment (34). Moreover, Green Synthesis Derived CdS QDs with *Camellia sinensis* leaf extracts arrested lung tumor cells cycle and decreased cell viability (35). In addition they showed that leaf extract-mediated CdS QDs inhibited pulmonary infection-related gram positive *Streptococcus pyogens* and gram negative *Serratia marcescens in vitro* (36). Besides, Zhao et al. reported that nitrogen-doped carbon QDs (NDQDs) generated from diethylenetriamine (DETA) and D (+)-Glucose monohydrate had specific antibacterial activity against *Staphylococcus* by inducing the rupture and integrity loss of cytoplasmic membrane of methicillin-resistant *Staphylococcus aureus* (37).

Photodynamic therapy (PDT) is a novel and innovative method for treating tumor in which a photosensitizing agent is administered and then exposed to visible or invisible light (38). Hsu et al. reported that Renilla luciferase-immobilized QDs-655 was employed for bioluminescence resonance energy transfer-mediated PDT to efficiently generate ROS, *in vitro* killing tumor cells and *in vivo* inhibiting tumor growth (39). Additionally, Choi et al. found CdSe/ZnS QDs irradiated by ultraviolet A/B inhibited the viability of lung tumor cells and induced cell apoptosis, suggesting that UV irradiation enhanced the efficacy of QDs in photodynamic cancer therapy (40). Besides, Liu et al. encapsulated BPQDs with exosomes (hEX) and found that hEX@BP showed evident tumor cells inhibition in a mice subcutaneous lung cancer model. When combined with photothermal therapy, hEX@BP got a more evident inhibitory effects against tumor cells (41), demonstrating great potentials for clinical applications.

Pulmonary arterial hypertension (PAH) is known as hypertension with high blood pressure in the lungs and primarily affects the pulmonary vasculature (42). In this regard, Zhu et al. reported that amorphous nano-selenium QDs (A-SeQDs) increased cellular tetrahydrobiopterin to protect against PAH through reuniting endothelial nitric oxide synthase (43). Specifically, A-SeQDs not only enhanced nitric oxide production and intracellular BH4 levels, but increased the activity of dihydrofolate reductase in lungs, above of which upregulated pulmonary arterial remodeling. The role of dihydrofolate reductase in preventing PAH was verified by gene knockout mice. In addition, clinical studies showed that the reduced tetrahydrobiopterin and selenium in the blood of patients with PAH confirmed the role of dihydrofolate reductase in the protection from pulmonary arterial hypertension.

## Toxicities of QDs in Lung

**Figure 1:**
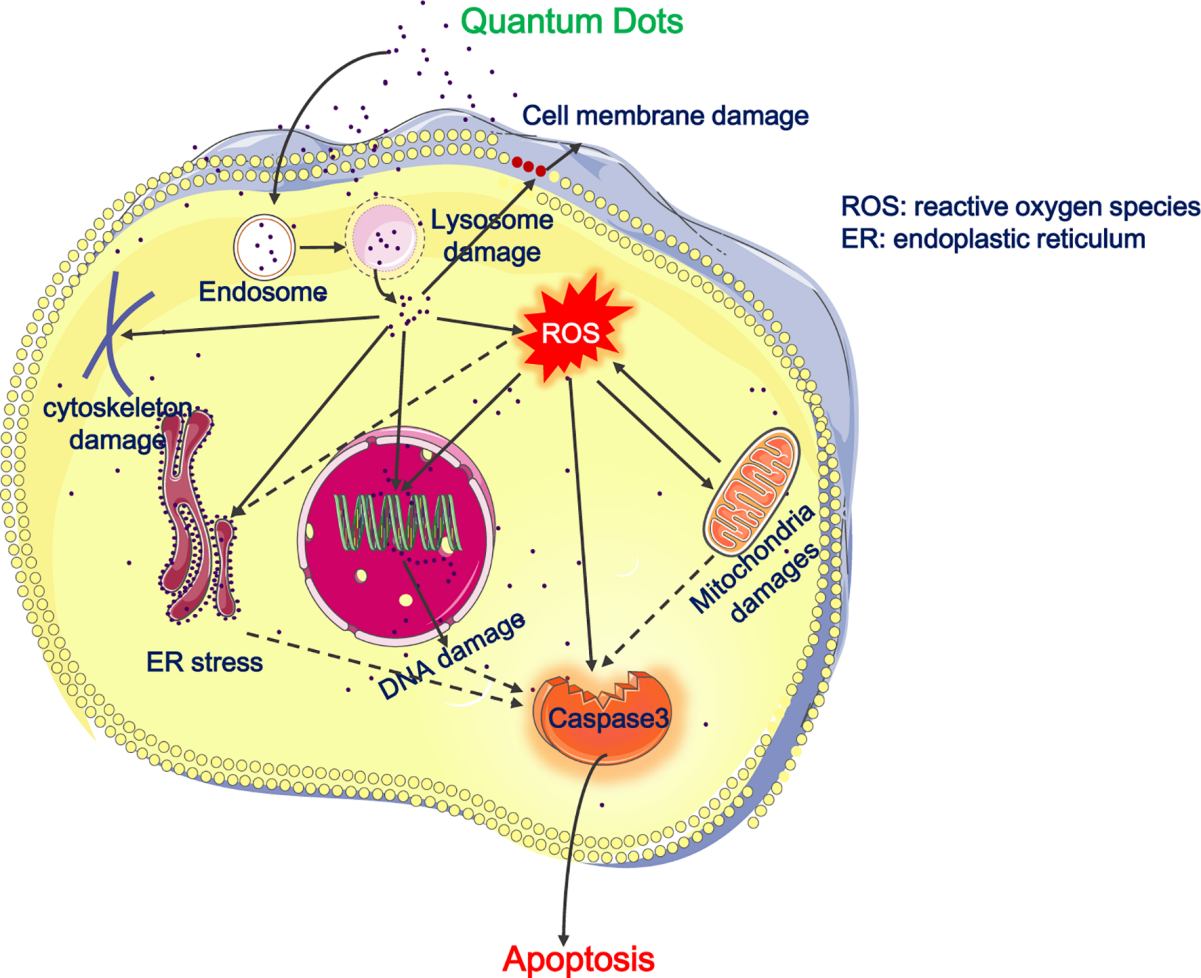
Cytotoxicity of QDs on the respiratory system. The picture illustrates that QDs entered the endo-lysosomal system and then are released into cytoplasm, resulting in cell membrane damages, depolymerization or disruption of cytoskeleton, ROS generation, mitochondrial damages, ER stress, DNA damage, and apoptosis. Besides, QDs-induced ROS promotes mitochondrial damages, ER stress, and apoptosis. Full lines represent the situation demonstrated and dotted lines represent our deductions.

The toxicity concerns regarding QDs are mostly connected with their chemical compositions, especially heavy metal ions in the core of QD such as Cd and Hg which might be released upon endocytic uptake into the cytoplasm of cells (**Figure 1**). However as for all nanoparticles with an extreme high surface to mass ratio, the surface reactivity of QDs is of toxicological concerns and accordingly often modified by surface passivation, e.g., *via* PEGylation.

### *In Vitro* Pulmonary Cytotoxicity

CdTe QDs are a kind of widely employed QDs in biomedicine, and their safety concerns people most. Zheng et al. presented findings about effects of CdTe QDs with different particle sizes on normal human bronchial epithelial cells (44). Acute exposure to CdTe QD induced dose-dependent cytotoxicity and carcinogenicity in BEAS-2B; chronic exposure induced BEAS-2B cell transformation including enhancing cell migration. They further examined the cellular response at the proteome level treated with CdTe QDs. 520Q with 520 nm emission maximum and 580Q with 580 nm emission maximum treatment changed cells proteome greatly in a very similar magnitude. Pre-treatment of cells with glutathione impeded the different upregulated/downregulated proteins and blocked cell death, indicating that ROS mediated QDs-induced cytotoxicity (45).

Besides, Chen et al. present the cytotoxicity of InP/ZnS QDs decorated with NH_2_, COOH, OH in human lung cancer cell and alveolar type II epithelial cell (46). High doses of all three QDs decreased the cell viability, causing intracellular ROS generation and cell apoptosis. Additionally, COOH QDs and NH2 QDs were more toxic than OH QDs, suggesting that surface decoration and concentration of InP/ZnS QDs should be optimized well for therapeutic purpose or biological imaging.

Stan et al. reported effects of Si/SiO_2_ QDs on human lung fibroblasts MRC-5 cells. They found Si/SiO_2_ QDs increased ROS and malondialdehyde (MDA) levels and decreased glutathione contents, suggesting that Si/SiO_2_ QDs’ cytotoxicity on human lung fibroblast was caused by disturbing cellular homeostasis (47). Furthermore, they found Si/SiO_2_ QDs induced MRC-5 cellular membrane disruption, changed cell morphology as actin filaments disrupted. Besides, matrix metalloproteinase (MMP)-1 and MMP-2 and also MMP-9 activity decreased which resulted in an unbalanced extracellular matrix turnover, for which MMPs might be risk factors of pulmonary fibrosis as SiO_2_ is a well-known harmful silica agent closely related to silicosis (48).

Because of GQDs biocompatibility and safety, GQDs-related nanomaterials received much more attention. Yuan et al. explored the cytotoxicity of GQDs decorated with COOH, NH2, and CO-N (CH3)2 in A549 cells. By employing trypan blue and thiazoyl blue colorimetric (MTT) assays in order to detect cell viability or flow cytometry analysis to detect cellular apoptosis or necrosis, they found all three GQDs had excellent biocompatibility and low cytotoxicity independent of chemical modifications (49). However, there are also some reports about the harmful effects of GQDs. Tian et al. explored the effects of hydroxyl-decorated GQDs (OH-GQDs) on A549 (p53^+/+^) and H1299 (p53^−/−^) cells. They found OH-GQDs enhanced intracellular ROS generation, led to cell cycle arrest and cells senescence (50). Besides, Xu et al. reported that aminated graphene GQDs (AG-QD) accumulated in rat alveolar macrophages nuclei, further resulting in nuclear damages and DNA cleavage. The detailed mechanisms were that AG-QD induced oxidative damage mediated by directly contacted *via* H-bonding and π–π stacking between AG-QD and DNA and promoted the upregulation of caspase genes (51) (**Figure 1**).

### *In Vivo* Pulmonary Tissue Toxicity

There are several reports indicating QDs depositions resulting in lung tissue damages.

Roberts et al. reported that CdSe/ZnS QDs led to lung abnormities accompanied with increased lactate dehydrogenase, lung injury parameters and albumin. The injury was at its severest at days 7 and 14 after inhalation. QDs dose had a positive correlation with the lung damage severity (52). Also researchers started to get interested in QDs effects on lung mechanics. Scoville et al. reported that amphiphilic polymercoated CdSe/ZnS QDs affected lung mechanics in A/J mice only but not C57BL/6J through using forced oscillation. Besides, they found significant inverse relationships between lung glutathione levels and the lung mechanics by measuring Resistance and Tissue Damping in QD-treated mice (53). Tang et al. reported that CdSe/ZnS QDs decorated with cationic polydiallyldimethylammonium chloride showed acute severe toxicity because of pulmonary embolism. All QDs caused injuries in specific tissues such as lung and liver after acute or long-term exposure, however, the injury degree was determined by their surface properties (54). Yang et al, found that 218 genes were significantly differentially expressed in the lung after ZnO QDs treatment by RNA sequencing. Related signaling and pathways mainly included cell DNA replication, peroxisome proliferator-activated receptor (PPAR) signaling, retinol metabolism, p53 signaling pathway and cellular senescence (55).

The surface modification of QDs could influence *in vivo* toxicity and the biological behavior. In this context, Li et al. explored the *in vivo* toxicity and distributions of InP/ZnS QDs decorated with COOH, NH2, and OH, in BALB/c mice after being intravenously injected. They found there were no evident histopathological abnormalities in all mice tissues after exposure to these three QDs. However, high dose of QDs-NH2 and QDs-COOH resulted in acute inflammation of the whole body but not QDs-OH. In addition, high-dose QDs-COOH induced mice death and slight liver function alternations (56). Moreover, in BALB/c mice Lin et al. also explored acute toxicity of the above three InP/ZnS QDs with aerosol inhalation. All QDs deposited in the lung, but the amount of QDs-OH was the most abundant possibly because of its largest size in aqueous solutions. Similarly, there were no histopathological conditions in the main mice organs. However, QDs-NH2 led to obvious hyperemia in alveoli septum (57). Additionally, Rehberg et al. reported that amine-modified CdSe/ZnS QDs (PEG), but not carboxyl-CdSe/ZnS QDs (PEG), accumulated in the postcapillary venule vessel wall and increased ischemia–reperfusion-induced leukocyte transmigration in postischemic heart and skeletal muscle (58). Therefore, the surface chemistry of QDs should be given adequate attention to for their biomedical applications.

## Immunological Effects of QDs

QDs immunological effects have been studied at the cellular level, organs and the whole body as well in mice. After exposure, QDs are recognized and “swallowed” by lung tissue cells such as epithelial cells and immune cells. Generally, QDs would pose damages to them and induce inflammatory responses, which would recruit innate leukocyte cells (e.g., macrophages and neutrophils) and also adaptive immune T cells.

Several literatures showed the potential of QDs to modulate lung epithelial cells, fibroblast cells or alveolar macrophages inflammatory response, like the activation of proinflammatory signaling or the promotion of cytokines release *in vitro*. For example, Stan et al. reported that Si/SiO_2_ QDs enhanced the production of nitric oxide, interleukin-6 (IL-6) and IL-8 expressions in human fibroblast MRC-5 cells (47). Besides, in cellular levels Lee et al. showed TOPO-PMAT CdSe/ZnS QDs induced expressions of neutrophil chemokines Chemokine (C-X-C motif) ligand (CXCL) -1, CXCL-2, IL-6, IL-12, and other proinflammatory factors in mice tracheal epithelial cells, alveolar macrophages, and bone marrow-derived macrophages (59).

*In vivo*, Ho et al. discovered intratracheal instillation of QD705-COOH induced acute neutrophils infiltration, interstitial lymphocytes infiltration, and a granulomatous reaction with cytokines, chemokines, and metalloproteinase 12 expressions (60). Furthermore, they found QD705-COOH-induced IFN-β expression might be dependent on Toll-like receptor pathways which was dependent on Toll/interleukin-1 receptor domain-containing adapter protein (61).

Besides, Roberts et al. showed that the treatment of CdSe/ZnS induced rat pulmonary inflammatory chemokines, increased innate immune cells (polymorphonuclear cells and alveolar macrophages) and also adaptive immune lymphocytes, indicating the leading to strong immune responses of CdSe/ZnS QDs (52). McConnachie et al. also showed that TOPO-PMAT CdSe/ZnS QDs induced the releasing of CXCL-1, GM-CSF, MIP-1α, and MIP-1γ, and in mouse bronchoalveolar lavage fluid (BALF), and also increased neutrophils infiltration but not alveolar macrophages. They found significantly inverse association between lung tissue cytokines levels, glutathione and BALF neutrophils and deposited pulmonary Cd QDs, indicating decreased glutathione might be the reason of QDs-induced lung inflammation (62). Also, Scoville et al. reported similar findings in NOD/ShiLtJ or NZO/HlLtJ mice (63).

Except for research about QDs direct treatment on lung, Scoville et al. explored the combined effects of house dust mite (HDM) and TOPO-PMAT CdSe/ZnS QDs on allergic airway disease (AAD) of C57BL/6J and A/J mice. Compared with C57BL/6J, they found that HDM plus QD group of A/J mice had more significantly enhanced levels of BALF IL-33 than that in HDM and saline controls. Moreover, A/J mice had greatly more innate lymphoid 2 cells (ILC2s) cells than C57BL/6J mice. ILC2s in A/J mice lung were negatively related to lung glutathione and resident macrophages with high MHC-II, and positively related to resident macrophages with low MHC-II, suggesting QDs could aggravate HDM-induced the development of AAD by recruiting ILC2s and increasing selected cytokines production (64).

In the above literatures, QDs’ proinflammatory and immune responses activation roles have been reported. However, there are also reports that QDs negatively regulate inflammation or immunological responses. Firstly, Volarevic et al. showed that GQDs significantly inhibited concanavalin A-induced mouse hepatitis. Specifically, GQDs decreased both apoptosis and autophagy in liver tissue which were associated with the reduced liver T cells producing IFN-γ and a serum IFN-γ decrease (65). Also, recently, Lee et al. reported that GQDs effectively alleviated dextran sulfate sodium-mediated acute and chronic colitis model by inhibiting TH1/TH17 polarization, switching macrophage M1 polarization to M2, and enhancing intestinal regulatory T cell infiltration (66). However, whether GQDs also show anti-inflammatory effects on lung inflammatory diseases like AAD or bacterial or virus infection-induced inflammation and cytokine storm is still unknown and needs to be investigated in the future.

**Figure 2:**
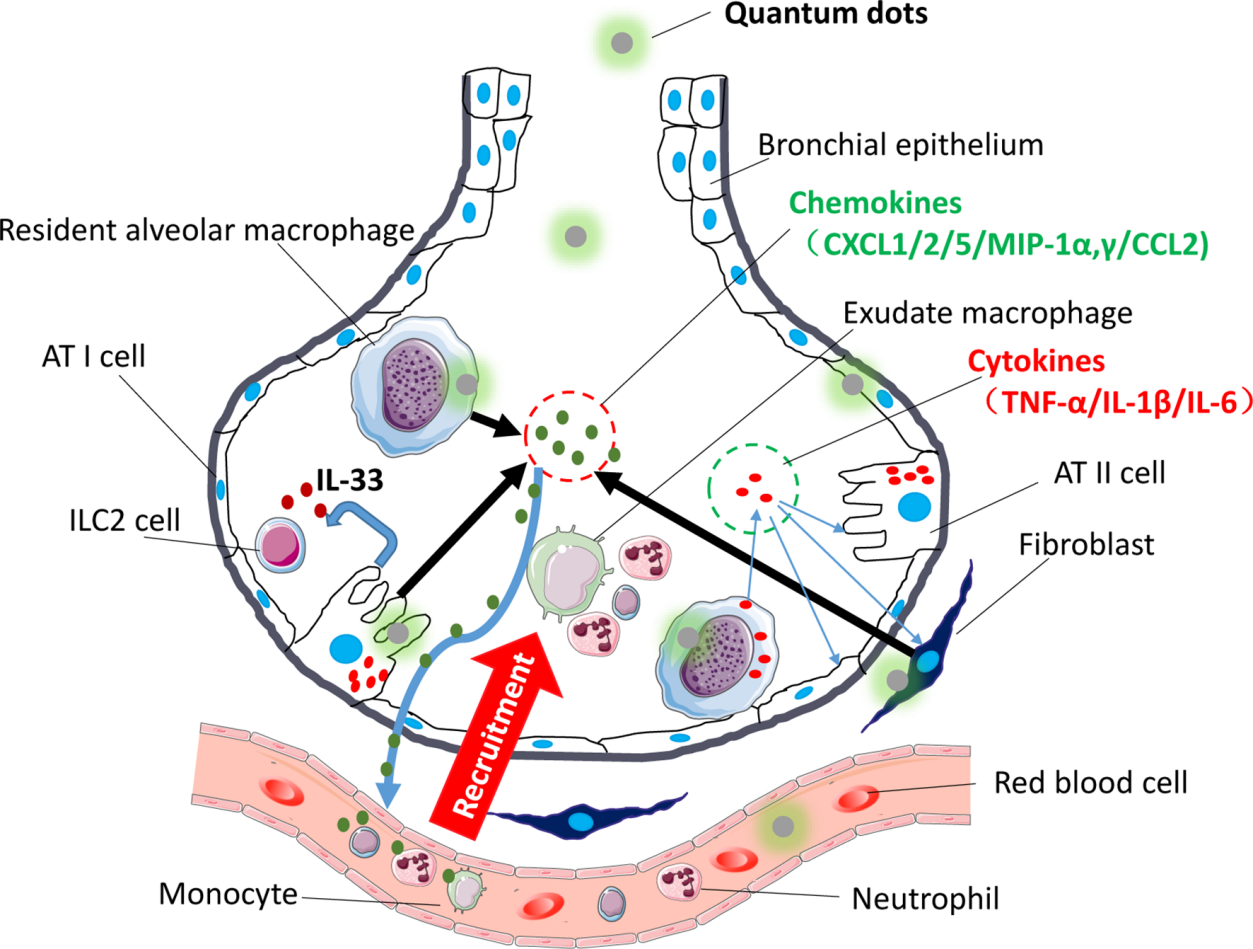
Immune modulatory effects of QDs on the respiratory system. The picture shows that after being exposed to QDs, resident pulmonary macrophages, lung epithelial cells, and fibroblast swallowed QDs, and then QDs would activate inflammatory-related pathway like TLR signaling, which promoted cells to release proinflammatory cytokines (TNF-α, IL-1β, and IL-6) and chemokines (CXCL-1/2/5/MIP-1α,γ/CCL-2). Sequentially neutrophils would be recruited firstly, and then monocyte and adaptive immune T cells would follow. Besides, QDs activated AT II cells and released IL-33 which activated ILC2 cells.

Based on the above, different kinds of QDs would induce respiratory inflammatory response, but the related further details or the underlying mechanisms need to be examined carefully. For example, QDs caused DNA damages in A549 and Beas-2B cell lines (67, 68), and DNA damages reagents-induced cells inflammation are dependent on Toll-like receptors 9 (TLR9) receptor or Cyclic GMP-AMP synthase (cGAS)-Stimulator of Interferon Genes (STING) pathway (69). Therefore it might be possible that QDs-induced cell damage also activates nucleic acid receptor TLR9 or cGAS-STING to mediate inflammatory responses. Alveolar macrophages include proinflammatory M1 type and anti-inflammatory M2 type (**Figure 2**). Whether QDs-induced lung inflammation could be attributed to excessive M1 macrophages activation and damaged M2 macrophages functions also needs to be explored in the future.

Collectively, employing QDs would perturb normal cell signals and cytoskeleton homeostasis, damage essential organelles, e.g., mitochondria and endoplasmic reticulum, and even activate related programmed cell death, which were mainly attributed to QDs’ heavy metal cores. Since pulmonary delivered particles are cleared from the lungs only very slowly and thus persist over a long period of time (70), the use of biopersistent materials such as QDs for diagnosis or therapy has to be balanced for its pros and cons.

## Conclusion

QDs have high potentials for biomedical applications in areas like bio-imaging, drug delivery, and diagnosis in the pulmonary system. For QDs to be realistically translated into clinical applications, issues such as pulmonary toxicity and immunological responses triggered by QDs should be addressed. Decreasing the toxicity of QDs for example by surface coating with more safe and biocompatible materials or replacing the heavy metal core, or the usage of low toxicity chemical cores such as Zn and graphene still should to be taken into account. In this mini review, we summarized the newest progress in the literature about the employment and the shortcomings of QDs for bioapplications to the respiratory system.

## Author Contributions

LR and LW performed literature search and prepared the first draft and displayed items of the mini review. MR and TS were involved in critical discussions of the content and display items and revising the draft. JZ and SC were involved in critical discussions of the content and displaying items, writing, and editing of the manuscript. All authors contributed to the article and approved the submitted version.

## Funding

This work was supported by the National Natural Science Foundation of China (81803183) and the Clinical research of Health and Family Planning Commission of Shenzhen Municipality (SZLY 2017024 to LW).

## Conflict of Interest

The authors declare that the research was conducted in the absence of any commercial or financial relationships that could be construed as a potential conflict of interest.

## Publisher’s Note

All claims expressed in this article are solely those of the authors and do not necessarily represent those of their affiliated organizations, or those of the publisher, the editors and the reviewers. Any product that may be evaluated in this article, or claim that may be made by its manufacturer, is not guaranteed or endorsed by the publisher.
